# Perceived expressed emotion in individuals with a first episode of psychosis from a south Asian background

**DOI:** 10.1111/eip.13542

**Published:** 2024-05-04

**Authors:** Amrita Ramanathan, Syed K. Miah, Lidushi Nagularaj, Hira Salman Sharif, Madiha Shaikh

**Affiliations:** ^1^ Research Department of Clinical, Educational and Health Psychology University College London London UK; ^2^ Division of Psychiatry University College London London UK; ^3^ North East London NHS Foundation Trust Rainham UK

**Keywords:** caregiving, EE, expressed emotion, first episode, psychosis, qualitative, schizophrenia, south Asian

## Abstract

**Aim:**

To explore perceived expressed emotion in the south Asian context for individuals with a first episode of psychosis (FEP).

**Method:**

Semi‐structured interviews were conducted with 16 service users experiencing a FEP to understand their experience of expressed emotion (EE) from their caregivers. Interviews were analysed using inductive thematic analysis.

**Results:**

Four main categories were identified: connection and support, understanding and awareness, boundaries and independence and context and influence. Factors influencing perceived expressed emotion such as acceptance, acculturation, warmth and expressions of love, communication and family values were identified. Findings highlight south Asian's experiences of being cared for, and their perception of EE, including warmth and connection as a strength and resource.

**Conclusion:**

The findings shed light on culturally specific EE within the context of FEP that can be considered when working with south Asian communities within early intervention services. Findings highlight the impact of navigating and negotiating bicultural identities and generational differences in EE in the British south Asian context.

## 1 INTRODUCTION

Expressed emotion (EE) can be defined as a set of attitudes, emotions and behaviours expressed by relatives about a family member. Families with higher levels of EE are understood to have high criticism, hostility and emotional over‐involvement (EOI). Studies have found that the risk of relapse for a mental illness is greater if families are over‐involved, critical and hostile (Brown et al., 1972; Vaughn & Leff, 1976). With respect to patient perceptions of caregiver EE (perceived EE), higher levels of perceived criticism are associated with lower levels of social functioning, higher levels of negative affect and negative schematic beliefs in psychosis (Onwumere et al., 2009) and prolonged high‐EE is associated with higher transition to FEP and higher levels of depression in those at risk of developing psychosis (Izon et al., 2021). Lower patient ratings of family cohesion and caregiver warmth are associated with greater psychosis symptom severity (Gurak & De Mamani, 2016). When examining EE in ethnic minority groups, patient perspective may be a better predictor of clinical outcome than standard measures of caregiver EE making it an important factor to investigate (Tompson et al., 1995).

Cross‐cultural literature demonstrates variation in EE, both in prevalence and in its relationship to patient outcomes (Bhugra & McKenzie, 2003; Cheng, 2002; Jenkins, 1992; López et al., 2009; O'Driscoll et al., 2019; Singh et al., 2013). Behaviours have been defined as pathological against UK norms, with EOI, being evidenced through over‐protective and self‐sacrificing actions, considered maladaptive in individualistic Western culture (Bhugra & McKenzie, 2003). Hence studies have found that levels of EE and their relationship to clinical symptoms and outcomes vary based on culture and ethnicity (De Mamani et al., 2007; Kopelowicz et al., 2002; Rosenfarb et al., 2006; Weisman, 1997) and a lack of ‘emotional over involvement’ from family members may be understood as a lack of care (Akhtar et al., 2013; Singh et al., 2013). Cultural differences in family involvement and support play a role in explaining mental health treatment disparities (Snowden, 2007). In diverse cultural groups, the predictive links of high EE and poorer outcomes are not uniform for example EOI may not necessarily be detrimental in all cultures. The effect of high EOI may be moderated by the unexplored dimension of warmth and high levels of mutual interdependence in kin relationships (Singh et al., 2013) and therefore need further investigation (Onwumere & Kuipers, 2021). In addition, characteristics such as ‘involvement’ and ‘self‐sacrifice’ in south Asian caregiving relationships may serve protective functions (Sharif et al., 2022). Such cultural variability points to the inherent complexity and need to critically examine the assumption that reducing EE within families is always appropriate and of clinical benefit for the patient.

There have been few studies exploring EE in south Asian groups living in a western context. Hashemi and Cochrane and Hashemi (1999) found a strikingly variant result within south Asian populations such that British Pakistani families had higher level of EE compared with British Sikh and White families who care for an individual with psychosis. However, EE did not predict psychosis relapse (Cochrane & Hashemi, 1999). Another study found baseline EOI was not associated with relapse at either 1‐ or 2‐year follow‐up among a North Indian community (Leff et al., 1990). Pakistani relatives showed higher levels of EOI and hostility as compared with many other cultures (Ikram et al., 2011). In most studies, south Asian perspective has been understood as part of a larger mixed ethnicity group; hence, it is difficult to capture specific narratives of south Asian community (Roach, 1992).

In recognition that (i) EE may capture cultural constructs rather than pathogenic traits, (ii) EE constructs may not translate across various cultures and (iii) there is limited understanding of perceived EE in relation to culture, there has been increasing call for studies to examine the cross‐cultural variability of EE (Domínguez‐Martínez & Medina‐Pradas, 2021; O'Driscoll et al., 2019; Sharif et al., 2022; Singh et al., 2013). To the best of our knowledge, this is the first study exploring perceived EE in south Asian families in the UK in the context of a first episode of psychosis (FEP). This study aims to expand on the existing literature by (i) examining EE within a specific cultural context. Using participant interviews and inductive thematic analysis, the study aims to deconstruct the concept of EE, explore perspectives of individuals with a FEP and their perception of EE within the British south Asian cultural context.

## 2 METHODS

This study received ethical approval by the Yorkshire and The Humber—South Yorkshire Research Ethics Committee (IRAS: 230098). All participants gave written consent to take part in the study.

### 2.1 Service user consultation

A panel made up of members from a London NHS trust patient experience service and a local grassroots SU led mental health organization in London provided feedback on the research proposal and NHS ethics application. This included two service users (SUs) with the early intervention service (EIS) from a south Asian background and they were particularly pleased that the study explored issues specific to south Asians. The interview schedule was piloted with three SUs from EIS to determine the order of the questions, whether they were understandable and fit for purpose and if the interview completion time was appropriate. Based on feedback, instructions were simplified, and interview length would be monitored to a maximum of 90 min.

### 2.2 Participants

A total of 16 participants were recruited from early intervention in psychosis teams between October 2020 to May 2022. A sample of 16 participants was sufficient to reach data saturation and serve the requirements of the objectives and research design utilized in this study (Vasileiou et al., 2018). Data saturation was identified when no additional themes were identified from interviews and at least one participant was recruited from the different services. Inclusion criteria included being under the care of the EIS service with a diagnosis of FEP for a period of at least 6 months to ensure that they met criteria for FEP; over the age of 18; English speaking and self‐identified south Asian heritage including the following countries Afghanistan, Bangladesh, Bhutan, India, Nepal, Maldives, Pakistan and Sri Lanka.

### 2.3 Data collection

A semi‐structured interview which explored perceived EE was conducted via Microsoft Teams and audio/video recorded (three were audio only). Interviews were no longer than 90 min to prevent fatigue and the average length was 19 min. All participants were eligible to receive £10 compensation for their time.

### 2.4 Interview schedule

The semi‐structured interview consisted of five open‐ended questions designed to capture perception of expressed emotion. The schedule followed the ethos of commonly used tools to identify EE, that is, the five‐minute speech sample (Gottschalk & Gleser, 1969), which use an exploratory approach to understand the nature of both positive and negative aspects of relationships. Participants were requested to think of a person they have a close relationship with when answering questions. This person could be (a) someone that you live with or have done in the recent past (b) a partner or relative (e.g., boyfriend/girlfriend/spouse/child/parent/sibling/niece/nephew/uncle/aunt/grandparent/grandchild and so on) (c) someone who would take a significant role in caring for you. Interview questions consisted of: what is your relationship with X like; has your relationship with X changed since contact with mental health services/early intervention team; what kind of aspects do you like about your relationship with X; what kind of aspect do you not like about your relationship with X and is there anything in your relationship with X that you would like to change/different. Interviewers were trained to prompt participants with open‐ended verbal cues. Interviews were conducted by the research team which included three trainee clinical psychologists. All interviews were recorded and transcribed using Microsoft teams and then formatted and stripped of any identifiable information.

## 3 ANALYSIS

Inductive thematic analysis (Braun et al., 2019) was used for identifying, analysing and reporting recurring patterns within the data to understand how patients perceive caregivers' EE. Themes were identified based entirely upon the data collected rather than on pre‐existing theoretical concepts (Pope et al., 2000). Inductive thematic analysis included five steps; (1) transcripts were reviewed, (2) initial core themes were identified using in NVivo (QSR International Pty Ltd, 2008), (3) codes were then grouped into core themes (4) themes, codes and interviews were reviewed by a trainee clinical psychologist, and a clinical psychologist/research supervisor both individually and jointly and (5) once a consensus was reached researchers created a document of the core themes and codes with examples and definitions.

### 3.1 Researcher's reflexivity

All researchers underwent a bracketing interview with an external clinical psychologist to the study with expertise in qualitative research to examine biases and prejudices that may inform the interview process and way themes are categorized and understood. This process highlighted how the researchers own experiences as a south Asian individual has potential influences on the interpretation and analysis of the themes. Furthermore, identifying as south Asian may bring certain cultural assumptions during the analytic process about how participants may feel towards their carer and what their expectations are of their carer. In addition, researchers maintained reflexive journals to continuously consider their position during interviews and analysis.

Once key themes were established, the research team consisting of four researchers reviewed the transcripts and further sharpened definitions of coding themes via consensus discussions and considered how the researchers identities inform the analysis.

## 4 RESULTS

### 4.1 Demographics

Sample demographics can be seen in Table 1. The average age of the participants was 30.8 years (SD = 10.9) with a range of 21–59 years. Main ethnicities included Pakistani (*n* = 4, 25.0%), Indian (*n* = 5, 31.3%) and Sri Lankan (*n* = 5, 31.3%), followed by Bangladeshi (*n* = 2, 12.5%). Participants were split relatively equally between female (*n* = 9, 56.3%) and male (*n* = 7, 43.8%). Majority of participants were born in the UK (*n* = 10, 68.8%), were single (*n* = 13, 81.3%;) and unemployed (n = 10, 62.5%;). Education level was mostly split between A‐level/vocational level training (*n* = 6, 37.5%;) and higher education or professional/vocational equivalent (*n* = 8, 50.50%;). The majority of participants spoke about a parent (*n* = 13, 81.3%), a few spoke about a partner (*n* = 2, 12.5%;) and their child (*n* = 1, 6.3%).

**TABLE 1:** Demographic information.

Age, mean (SD)	30.8 (10.9)
Range	21–59
Ethnicity *n* (%)	
Bangladesh	2 (12.5)
India	5 (31.3)
Pakistan	4 (25.0)
Sri Lanka	5 (31.3)
Country of Birth *n* (%)	
Bangladesh	1 (6.3)
India	1 (6.3)
Spain	1 (6.3)
Sri Lanka	2 (12.5)
UK	11 (68.8)
Gender *n* (%)	
Female	9 (56.3)
Male	7 (43.8)
Religion *n* (%)	
Buddhist	1 (6.3)
Christian/Catholic	4 (25.0)
Hindu	2 (12.5)
Muslim	6 (37.5)
None	1 (6.3)
Sikh	2 (12.5)
Relationship Status *n* (%)	
In a relationship (not cohabiting)	1 (6.3)
Married	2 (12.5)
Single	13 (81.3)
Education level *n* (%):	
A Level, vocational level (e.g., NVQ) 3 or equivalent	6 (37.5)
GCSE (5 or more grades A*‐C), vocational level (e.g., NVQ)	2 (12.5)
Higher education or professional/vocational equivalent	8 (50.0)
Employment status *n* (%):	
Apprenticeship	1 (6.3)
Employed full time	3 (18.8)
Employed part‐time	2 (12.5)
Unemployed	10 (62.5)

*Note*: *M* = Mean; SD = standard deviation, *n* = participant count.

### 4.2 Main findings

Table 2 displays an overview of the thematic analysis where four main categories were identified; (1) connection and support, (2) understanding and awareness, (3) boundaries and independence and (4) context and influence. Within these categories, 14 themes and 6 subthemes were identified. Table A1 in the supplementary material contains quotations from participants to illustrate the themes and subthemes.

**TABLE 2:** Summary of themes.

Categories	Themes and subthemes
1. Connection and support	Love and expressions of love Physical affection of care Humour and playfulness and a sense of safety Unconditional love
	Support Advice, wisdom and knowledge Kindness and listening, bonding and spending time Financial and practical support
	Mutual relationship
	Concern for carer's health
	Appreciation and gratitude for one another
2. Understanding and awareness	Awareness and understanding of mental health
	Fear of being rejected
	Open dialogue and communication
3. Boundaries and independence	Acceptance about carer
	Controlling, intrusiveness and protectiveness
	Negotiating change in relationships and arguments
4. Context and influence	Cultural and generational differences
	Family roles and dynamics
	Mental health

## 5 CONNECTION AND SUPPORT

### 5.1 Love and expressions of love

#### 5.1.1 Physical affection of care

This mainly presented in the context of women speaking about their relationship with their mother or daughter. For example, participant 1157 described physical affection ‘like she helped me with the cooking, and she shows me affection like gives me hugs and tells me to be there for me that nothing's going to happen to you. I'm here for you and she always tries to see the positive things in life, and she pushes me up to do well and things like that’ (1157).

#### 5.1.2 Humour and playfulness that creates safety

Humour and non‐verbal playfulness were used as a way of demonstrating closeness and love. There was a sense that carer's expression of love, affection and humour provided participants with a sense of safety and comfort. For example, participant 1141 described teasing and joking ‘mucking about (pinch on the cheek for example)’ as a way of showing love for one another.

#### 5.1.3 Unconditional love

Several participants spoke of unconditional love that their carer had for them. This seemed to be contextualized in the family roles and duties. Participant 1084 said ‘I'd say we have an unconditional relationship, unconditional love relationship in the sense like normal mother would have for the son’. Similarly, another participant described that his wife was unconditionally committed to the relationship and did not expect any money but just his love.

### 5.2 Support

Participants spoke about instrumental and emotional support they received from their loved one both when they were unwell or experiencing mental health difficulties and more generically.

#### 5.2.1 Advice, wisdom and knowledge

Some spoke about how they valued advice or knowledge about certain things from their loved one as it helped face their low mood and it helped them excel in their career and life. For example, participant 1050 said that the knowledge her carer shares ‘does come in handy’.

#### 5.2.2 Kindness and listening, bonding and spending time

Kindness, care and being heard were valued. Participants described feeling supported and valued when they were listened to by their carers. For instance, participant 1023 described her mother as ‘a really good listener’ and that her mother ‘always… make[s] time for [her] when [she] need[s] her’. Similarly participant 1057 highlighted dialogue as important and said ‘I say it's a pretty good relationship like I talked to her a lot about my problems and when I'm like feeling upset or like feeling down. She can always usually tell without me even come telling her’.

Participant 1023 described that she and her mother are kind to each other by expressing their desire to spend time with one another. Many participants described bonding and spending time together as a way of recognizing the support they had. Participants whose carer was a parent or a child spoke about spending time through activities such as shopping, travelling, playing sport, going to the cinema and talking with one another.

#### 5.2.3 Financial and practical support

Participants who had children appreciated that their carers supported them with childcare tasks like taking the kids to school, as such participant 1109 said ‘he's looking after children, plus he's looking after me as well so it's a lot of hard work, hard job for him’. Participants who identified as children to their parents appreciated the commodities and education that their parent was able to buy them; for example, participant 1014 spoke about how his father shows care by buying him food and clothing as well as ‘actively trying to get [his] medication changes’ by calling his care coordinator. Several participants described their carer's supporting them with medications and coordinating care for their mental health needs. Participants found this practical support with medications and wellbeing supportive.

### 5.3 Mutual relationship

Participants spoke about how their relationship with their carer was a give and take, and that not only is their carer present and supportive for them, but participants reciprocate care and support to their loved ones. Participant 1023 said ‘sometimes she goes through things and then [she is] there for her too’.

### 5.4 Concern for carer's health

Participants who had carers of an older age described worrying about their carer's health and at times the longevity of their carer's life. Participant 1061 spoke about how she ‘wished that [her mother] would look after her health’. Some hoped that their carer could take better care of themselves whilst others worried about their responsibility in ensuring their loved ones had a long life.

### 5.5 Appreciation and gratitude for one another

Participants generally described a sense of gratitude and appreciation for having their loved one present. For some participants, the gratitude for the care they received made them feel that they could not ask for more as they were satisfied with the kind of care they received. For instance, participant 1061 said ‘I'm grateful enough and, uh, I can't really ask for more. I wouldn't want my mother to do anymore that, you know, to do what she is doing, yeah’.

## 6 UNDERSTANDING AND AWARENESS

Participants spoke about their loved ones understanding and awareness of their mental health and several factors that may have facilitated this understanding, including the involvement of EISs, open dialogue and communication, patience and reassurance.

### 6.1 Awareness and understanding of mental health

Participants described initially finding it difficult to explain their mental health experiences to their loved ones. If carers were engaged with the EIS, they gained a better understanding and awareness of the participant's mental health needs. Many participants described increased understanding and patience as their carers learnt more about what they were experiencing and this in turn facilitated open and honest conversation, which helped participants to explain their mental health as talk about non‐illness related matters (‘I think the conversations she had behind the scenes with the medical teams and the therapist without me helped her to leave me alone sometimes and helped her to deal with it, and which again made the relationship better, allowed us to communicate or personal things rather than the medical stuff’. [P1007]). One participant shared that their carer learnt to be aware of their mental health signs and checking in with them by offering to go out for a walk. Others described this feeling of not having to explain their mental health to their loved one and this made room for other conversations that helped them to bond.

### 6.2 Fear of being rejected

Sometimes participants felt nervous about speaking with their loved ones about their mental health for fear of being rejected or blamed. One participant (1007) spoke about the fear of shame and judgement from the wider family and as a result asked her mother ‘not to tell relatives or anything except [her] brother’.

### 6.3 Open dialogue and communication

Participants generally found that their communication with their family member had improved since contact with EIS and specifically after receiving psychological interventions such as family interventions and carer specific interventions aimed at psychoeducation and wellbeing. There was a notable increase in trust and a non‐judgmental attitude from carers as they understood more about their loved one's mental health through increased communication. One participant described her mother's openness in seeking medical advice is ‘calming and … reassuring’.

Others described they wished to communicate more openly about personal matters beyond logistical factors; hoping that they would be able to share more about what was going on for them, but their symptoms including paranoid ideation and negative symptoms might be a barrier for them to engage.

## 7 BOUNDARIES AND INDEPENDENCE

Some participants talked about negotiating and hoping for changes in their relationship, whilst others were more accepting of the nature of their relationship with their family member. Participants also highlighted aspects they found difficult or unhelpful such as their family member being controlling, intrusive and protective.

### 7.1 Acceptance about carer

When participants spoke about their carer's qualities, some described a sense of acceptance of the relationship between their carer and them. A common feature with ‘acceptance’ was that participants felt that the unhelpful qualities in their relationship were not significant enough to want to change. As one participant described that the unhelpful qualities of her mother is ‘not something so significant that [she'd] say [she] want[ed] to change it’.

### 7.2 Controlling, intrusiveness and protectiveness

Controlling behaviours were linked to the lack of autonomy experienced and feeling frustrated and less close with their loved ones. Although, some participants could understand the intent surrounding concern and worry underlying controlling behaviours such as regulating money, monitoring calls and enquiring when they go out, they were not willing to accept this and experienced this as controlling and intrusive. Participant 1084 said ‘if I'm on the phone to someone she'll just come up and be like what's it about who my talking to? I get annoyed at sometimes because I don't want her to come up to me all the time and ask me what I'm doing wrong, or grow up or who am I talking to what's it about and stuff’. Expectations and demands on academic performance and career were reported to be an unsaid standard in south Asian communities which drove some controlling behaviours.

Some participants described experiencing their carer as overbearing when they were unwell.

In conjunction with intrusiveness some participants also described that their carer was protective of them when going out with friends or speaking people on the phone. Some described this as overprotective and intrusive whilst others described it as an acceptable and understandable level of protectiveness either because of their mental health difficulties or it was understood to be related to cultural norms. Some participants experienced this level of intrusiveness/protectiveness as a lack of trust from their carer and participants described wanting a sense of independence and increased trust in their relationships. Participants found it a hard balance to strike between gaining a sense of independence and recognizing that their carer's worry and questions came from an understandable place. Other participants hoped to share and talk about more personal matters, to be able to further build the bond and mutual understanding with their carer in the hope this will reduce some of these behaviours.

### 7.3 Negotiating change in relationships and arguments

Negotiating levels of independence and autonomy with carers sometimes resulted in arguments and disagreements. Many described this as a natural part of the relationship however, others found this unhelpful and disruptive to the relationship. Over time many had noticed that the negotiations in their relationship with the support of the early intervention (EIP) team helped facilitate better understanding and communication between them and their carer. A few participants talked about their responsibility to make changes such as participant 1023 spoke about wanting to be more motivated to listen to their carer and do household chores. Another person spoke about how the simplicity of the relationship in terms of his mother's awareness or understanding of mental health meant that she asked less questions which were helpful. Some participants spoke about how their carer provided them with the space to come to their own decisions. Participant 1115 spoke about how his partner would not stop him from drinking or smoking when they were initially married but told him that he should ‘look after’ himself. He noticed that his habits changed slowly without her demanding those changes from him.

## 8 CONTEXT AND INFLUENCE

This theme captured how the dyad related with one another in the context and influences of cultural and ethnicity, generational background, other family members, the influence of roles in the family and mental health.

### 8.1 Cultural and generational differences

Participants spoke about the cultural and generational differences that might cause conflict within their relationships. This was particularly highlighted in parent child relationships, for example, participant 1023 spoke about how her carer ‘grew up in a different time, so she's much more traditional’. Class and educational differences that made their understanding and priorities different from their loved one were also mentioned. This was not always highlighted as a negative difference between the dyads, as many participants stated that this as the context for the difference but did not wish to change that.

### 8.2 Family roles and dynamics

This captured the influence of other family members that either facilitated or impacted the dyad. Participant 1031 spoke about her other daughters speaking to each other helped increase understanding and support for her daughter who lived with her as her carer. This in turn improved their relationship.

Some also spoke about how the different roles in the family changed what was expected for one another. Participant 1119 highlighted the different expectations as the boy child in his family and that his parents were partial to him in comparison to his sister. Those who spoke about females described more household chores and support in terms of affection, love and care. Those who spoke about males spoke about other kinds of bonding including financial support, sport and education.

### 8.3 Mental health and relationship with voices

A few described how their mental health symptoms would impact their relationship with their loved one, such that they were more likely to withdraw from them. For some, the voices they heard were actively creating mistrust or distance between them and their loved one. For others, like participant 1119, the voices were representation of the critical comments they received from their carer regarding their sexual identity.

## 9 DISCUSSION

This study used an inductive thematic analysis to understand EE in the context of a FEP and within a British south Asian cultural context. Four main themes were identified: connection and support, understanding and awareness, boundaries and independence and context and influence.

Most participants expressed support and connectedness with their loved one. Expressions of love included physical affection and non‐verbal display of care, kindness, safety, humour and playfulness. Social support is associated with better quality of life and functional status in psychosis (Howard et al., 2000). These themes link with studies that have identified warmth as a protective factor in relapse prevention (López et al., 2004). Although participants did not use ‘warmth’ explicitly to describe the carer's actions, love, support and care were used. Warmth may also have been related to better communication and understanding that was often found to be facilitated by the involvement of EISs. The common omission of warmth and positive regard from the literature means that little is known about the protective aspects of relationships, how they may interact with, and mitigate the impact of, negative aspects of EE in culturally variant ways (Bhugra & McKenzie, 2003; Singh et al., 2013). Indeed, warmth was shown to be protective against symptoms of psychosis for Mexican Americans, whilst not relating to the course of illness for Anglo American families (López et al., 2004).

There were some reported differences in expressions of love and support between female participants who spoke about their mothers and male participants when speaking about their fathers. When female participants spoke about their mother's care, they mentioned support in household chores and physical affection such as hugs and kisses. When male participants spoke about their fathers, they commonly mentioned the practical financial support and material support such as buying clothes. This may be partly due cultural norms around women expressing care with physical acts. A study exploring fathering behaviours and experiences among UK south Asian men similarly found that ‘income‐earning and material provision was consistently identified as an integral part of being a father across respondents from all four religious and ethnic groups (Bangladeshi Muslims, Pakistani Muslims, Gujarati Hindus and Punjabi Sikhs)’ whereas it was more common for mothers to hold personal caregiving tasks like cooking for the child (Salway et al., 2009).

Understanding and awareness were another prominent theme. Many participants expressed appreciation for their carer's increased understanding and awareness that was facilitated through the EIP services and psychological interventions. A qualitative study similarly highlighted that carers in ethnic minority groups report an increased understanding and awareness of their loved one's mental health through the support of EIS (Lavis et al., 2015). Additionally, these findings contradict some existing literature, for example one study highlighted that many south Asians found that services were not adequately addressing their needs and involving support from their family system (Bowl, 2007). Yet it should be noted that EIP services have evolved in the last decade to incorporate support for caregivers and provide tailored interventions targeting psychoeducation and wellbeing (Claxton et al., 2017) that have been shown to be helpful (Petrakis et al., 2014; Shaikh et al., 2022).

Fear of rejection, not only from their carer but the larger community was evident in the findings. This largely relates to the stigma that many individuals with psychosis experience from wider family and services among British south Asians (Vyas et al., 2021). The study indicated intersecting stigmatized characteristics such as race, religion and socio‐economic status. Like acculturative stress, the pressure to assimilate and integrate into one's community may result in significant stress, which may impact on mental health functioning (Rastogi et al., 2014).

Prominent subthemes within the theme of boundaries and independence included acceptance and negotiating carer's involvement. Many participants described being grateful for their carer's support and intentions. However, they also expressed frustration with lack of boundaries or autonomy in the relationship. Some reported minimal intrusiveness or controlling behaviours from their loved ones, whilst others mentioned this was a regular part of the relationship. At times carer's involvement experienced as intrusive, controlling or protective was in response to participant's mental health, whilst at other times participants mentioned that their carer has always been involved in this way before the development of FEP, alluding to a cultural norm. This might be indicative of south Asian family values and responsibility of care. Existing literature has identified that in some cultures a lack of ‘emotional over involvement’ from family members may be understood as a lack of care (Akhtar et al., 2013; Singh et al., 2013). A study investigating south Asian carers of people with dementia identified south Asian carers held more ‘traditional caregiving ideologies’ compared to White carers; this ideology was seen as natural, expected and virtuous (Lawrence et al., 2008).

It is important to note that there was a large variation in how participants described carers involvement and communicated the boundaries in the relationship. It may be that the perception of these carer qualities varies depending on several factors (i.e., level of controlling, protective or intrusive behaviours, the individual's own mental health, level of acceptance, acculturation and intergenerational differences). This is consistent with the idea that the intrusive elements of emotional over‐involvement are linked to relapse and worse clinical outcomes (Singh et al., 2013) and this relationship appears to apply in the context of British south Asians experiencing a FEP. Factors such as acculturation and assimilation might have an impact on the perception of involvement versus intrusiveness and needs to be further explored in the cultural context.

It may be that acculturational differences determine how dyads communicate and negotiate. For example, one study investigated the experiences of parents who immigrated from India, Bangladesh and Pakistan to United States. They found that acculturative stress experienced by the immigrant parents lead to unrealistic expectations for their children in the hopes deter their children from the same financial or educational struggle that parents may have faced (Bhattacharya & Schoppelrey, 2004) as was evident in our findings. This also fits with intergenerational trauma and how adverse experiences or limited opportunities can impact on parenting style, which can lead to unrealistic expectations for children (Lang & Gartstein, 2018). Acculturative stress and migration have been largely associated with high rates of mental health disorders among south Asian migrants (Karasz et al., 2019).

Another study found that when perceived external acculturation expectations contrasted with Muslim minority youth's personal acculturation preference this resulted in higher levels of stress and less adaptation (Kunst & Sam, 2013). The study also found that depending on the level of perceived external acculturation the types of acculturation strategies changed (i.e., integrate, assimilate or separate). In our study, when participants had different expectations for themselves around their cultural practices compared to their parents, pressure to acculturate resulted in frustration and an attempt to negotiate an acculturation strategy.

The varied perspectives of boundaries and independence can be attributed to a range of sociodemographic factors such as gender roles, language, economic mindset, family role, class, generation, roles associated with age and religion (Robinson, 2005). This links with prior research that identified differences in EE levels between two cultural similar groups British Pakistani and British Sikhs (Cochrane & Hashemi, 1999). This study supports the notion that there are varied perspectives on caregiving constructs between communities that are considered culturally similar. It further emphasizes the need for research and clinical practice to consider the nuanced intersectional identities that make subcultural experiences unique.

Acceptance can be a product of collectivist interest. For example family cohesion may be particularly protective for ethnic minorities (Gurak & De Mamani, 2016); hence, acceptance of carer's behaviours may be a result of not wanting to disturb the family system. Typically, Asian families often function as a unit, ‘parental obligation and family pressure can take precedence over autonomy with resultant emotional conflict’ (Ekanayake et al., 2012). As a result, young adults may have the stress of negotiating their ‘dual identity’, one at home and one outside, which may impact mental health (Rastogi et al., 2014). In the context of being a south Asian living within the UK, bicultural identity will also impact this dynamic and collectivist values.

### 9.1 Strengths and limitations

As participants were recruited through EIS the sample was biased to a certain level of cognitive and mental functioning, especially as those with internet or phone access were more contactable and engaged with the study. Although participants were told that their participation was voluntary and would not affect their clinical care, it is more likely that participants had more positive comments about the services and the role that the service played in facilitating communication with carers.

The English language inclusion criteria were applied due to limited funding resources to allow for the use of interpreters and translation. This limits exploration of caregiving constructs characterized in non‐English speaking communities; a community that is significantly underrepresented in research but a large part of the population who may identify as south Asian.

Qualitative and exploratory nature of the study made it difficult to ascertain the influence of symptoms of psychosis on participants perceived EE. For example, if participants found caregiving behaviours unhelpful, it was difficult to understand to what extent with was influenced by the voices or paranoid beliefs or if participants felt like this dynamic was beyond symptoms of psychosis. This is important to consider as externalizing participant's perceived EE to the symptoms of psychosis avoids pathologizing the patient and carer relationship. A possible consideration would be to examine the dyads perceptions to help understand the context of symptoms. Furthermore, the bracketing approach was utilized at the beginning of the interview process. Although reflexivity was demonstrated throughout the analysis process it may have been helpful to consider bracketing interviews at different stages of the analytic process.

### 9.2 Clinical and research implications

This study highlights the perspectives of individuals experiencing a FEP, which has been minimally examined in the context of EE and south Asian communities. Existing literature on EE has highlighted the need to better understand this construct from a cultural lens. Furthermore, the National Institute for Health and Care Excellence (Excellence, 2014) highlight the need to research whether culturally adapting family intervention for schizophrenia would facilitate engagement; thereby improving clinical care and reducing relapse/readmission rates for minority ethnic groups.

## 10 CONCLUSION

The findings from this study first shed light on a culturally specific account of EE within the context of FEP and can be considered when working with south Asian communities within EIP services. It implies that in this sample British south Asian caregiving experiences and perception of EE includes warmth and connection as a strength and resource and is influenced by acceptance, acculturation, communication and family values. The study suggests that south Asian caregiving experiences in the UK are complex and that multiple intersectional identities shape individual caregiving experiences in the context of biculturalism. Findings also highlight the impact of navigating and negotiating bicultural identities and generational differences in EE in the south Asian context. Future studies should further explore the impact of perceived EE on clinical symptoms and long‐term clinical outcomes as well as the relationship between perceived EE and caregiver EE utilizing dyads.

## FUNDING INFORMATION

The authors have no funding to disclose.

## CONFLICT OF INTEREST STATEMENT

The authors declare they have no conflict of interest.

## Supporting information


**TABLE A1.** Quotations illustrating themes and subthemes.

## Data Availability

The datasets generated during and/or analysed during the current study are available from the corresponding author on reasonable request.
