# Supplementary material for: Perceived expressed emotion in individuals with a first episode of psychosis from a south Asian background
Source: Early Interv Psychiatry. 2024 May 4;18(12):991–1000. doi: 10.1111/eip.13542 (PMC11625530; doi:10.1111/eip.13542)
Supplement: Supplementary file 1 — TABLE A1. Quotations illustrating themes and subthemes. [file EIP-18-991-s001.docx]

**Table 3.** Quotations illustrating themes and subthemes

| **Categories** | **Themes and subthemes with quotes** |
| --- | --- |
| **1. Connection and Support** | **Love and Expressions of Love**  ***Physical affection of care; Humour and playfulness and a sense of safety; Unconditional love***  “like she helped me with the cooking and she shows me affection like gives me hugs and tells me to be there for me that nothing's going to happen to you. I'm here for you and she always tries to see the positive things in life, and she pushes me up to do well and things like that.” (1157)  “Yeah, I like to play kind of like jokes on my mum, Uhm, just like mucking about really, I sort of just like pinch her, that kind of thing. (laughs) I usually pinch her on her cheeks or something like that or like arm. Yeah, I would say that that we are quite like playful in that sense like kids and so yeah and she just sort of reacts and I kind of like that reaction. So yeah …… she reacts back in like a playful way as well. She would just be like go away or like in Tamil she would say that and so it's just like a way we, just like, I guess not communicating but just have fun really.” (1141)  “I like being with her. She makes me laugh so I enjoy that she's quite funny. She’ll tell me jokes about her friends, or she'll say something or describes somebody funny. So she's quite nice. Sometimes I laugh and say depends on what level my mood is in… uh, there's a lot there. There's a lot of affection there, nice, I feel kind of quite safe with her.” (1031)  “I'd say we have an unconditional relationship, unconditional love relationship in the sense like normal mother would have for the son” (1084) |
|  | **Support**  ***Advice, wisdom and knowledge; Kindness and listening, bonding and spending time; Financial and practical support***  “ Well, he's very supportive of me. Yeah, he's very supportive. Uh, he's very helpful is quite knowledgeable, so he always likes to share knowledge with me, on different things. And knowledge does come in handy as well” (1050)  “We were just being really kind to each other. And then even when we came home and I was like, I really like spending time with you movie shopping and she was like yeah she, she always says that about me anyway, She's always like, yeah, you're really good company and that when we go out she's like, yeah, you're really good company. But this time I felt like I just felt a lot closer to her. I'm not sure why, maybe because I started doing my wedding shopping as well so it kind of hit us both that OK yeah like it happened. It's all happening so. I bought a few things for my wedding and yeah, like I think we kind of got a bit closer over that cos we were choosing other things together and stuff like that.” (1023)  “ I'm just because I said I I'm quite close to her. I had discussed like about things with her, but like my future, my past and the present and and I feel like we like bond a lot. So I think that's probably like a major reason why I feel like close to her.”(1057)  “ We do things together, right? We kind of sit and we talk together we’ll share things together. We'll have dinner together and like there's a lot of like the family things that I enjoy doing with her and she'll find out things to do right and then she'll sort of like you know, say to me there's this happening in barking shall we go there? Shall we do this? Shall we do that? It makes a lot more that she makes a lot more effort with me and that I like that.” (1031)  “Uhm, recently.. I say he’s looking out for me in terms of like my medication that I’ve been taking, so he’s been calling up my care coordinator to help me get it changed because I’ve gained a lot of weight on it. So he’s conscious that, about my health as well. So he’s been looking out for my health. Hmm, yeah, he’s been actively trying to get my medication changed as well, which I should be doing myself, but he’s just taking a care..taking care of it for me…” (1014)  “Uhm, I’ll say he’s very caring as well, like, he always looks out for me. He always buys my favourite food or gets me clothes and like always looks out for me and provides for me as well like and. Yeah, I think that’s it.” (1014)  “Yeah , he supports me a lot and I'm not able to do my things and all, so he does for me and especially my.. I was a good cook, cooking food and so now I'm not able to do so. He supports me with that so he's like he's looking after children, plus he's looking after me as well so it's a lot of hard work, hard job for him.” (1109)  “it’s compromising, UM, you know, in different aspects of, for example, like we share our duties like when I have a baby. So like when I look after the baby so we have one night when she look after them. Then one night I look after like that.” (1157)  “ I think I've touched on everything, but she's a really good listener. She always, you know, make time for me when I need her. Uhm, like just after I got ill when I came back home she'd always make me spend time with her like in her bedroom like, uhm, just before I go sleep. And she’d always call it. Like she’d be like yeah, we need to have a meeting in my room. She’d always call a little meeting.” (1023)  “Uhm, I say it's a pretty good relationship like I talked to her a lot about my problems and when I'm like feeling upset or like feeling down. She can always usually tell without me even come telling her. So I think we have a pretty good relationship. Uhm, yeah, we spend a lot of time together. And UM. Yeah, like, I listen to her advice and she gives me a lot of advice as well, so. Yeah, I’d say we have a good relationship.” (1057) |
|  | **Mutual relationship**  “Yeah, it's generally a good relationship and I'm just trying to think of aspects. Uhm? I think we're pretty understanding towards each other as well, because sometimes she goes through things and then I'm there for her too….” (1023)  “there’s marital problems between her and my dad sometimes. And there's like arguments so like, the way like I have leaned on her for support when I wasn't unwell, like she's kind of lent on me and my sister for support in that kind of thing, so it's like. Uhm, I like that we can talk about those things and just kind of like. Uhm, be there for one another” (1010)  “Uhm… I would be more caring and more loving and supportive and being there for her rather than her always being there for me. I would um be able to reciprocate those conversations, and I know she wants to talk to me about that stuff and I'm having to shut it down or leave the room and. So that makes the guilt difficult.” (1007) |
|  | **Concern for carer's health**  “I wish that she would look after her health… yeah ultimately I worry about her, you know, it's her.. health, uh.. well being, uh, you know, her life. So you know it would ultimately affect her.” (1061) |
|  | **Appreciation and gratitude for one another**  “No, not really. I mean, you know the fact that she's there is, you know, I'm grateful enough and, uh, I can't really ask for more. I wouldn't want my mother to do anymore that, you know, to do what she is doing, yeah.” (1061)  “I think it's kind of rare to find someone that fully supports you and everything that you do come apart from your family, sometimes with certain families you don't even get that. So I'm just grateful that like we have like supportive parents and someone there to care for us. So I guess in that sense, yeah. Yeah, that sense. It's just nice to have that person there just anchors us as people.” (1141) |
| **2. Understanding and Awareness** | **Awareness and understanding of mental health**  “I think they kind of like speak to each other a lot more right there. I'm like not a topic, but I can't find another word, but uhm, say you know when they meet with each other they talk about me. They will sort of like you know, say what's happening so they're getting bit more understanding and because I'm able to, right, put things in as well, right? When [care coordinator] says to me my appointments and so they are, they have checked. There are a lot more patient or a lot more understanding of me so they will listen to me more and they won't , they won't make me feel well, it's not bad, it's not happening.” (1031)  “Ohh yeah and just generally how I'm feeling or if I'm feeling a bit withdrawn he might ask are you ok? Something like that. Yeah a bit more aware of the yeah it's like the symptoms of mental health problems” (1123)  “Yeah, I think it's quite good, isn't it, just to be able to pick up on things. Like maybe sometimes I might be internalising some things and not thinking about it, even though I'm probably all right at the time. It's just good to have a like a check just in, just in case I'm not ok..which is quite nice.” (1023)  “I think she was very lost at the beginning and didn't really uhm understand what was going on. Very nervous and scared. The treatment team really helped her calm down and they will have their phone heard. Have conversations about me just, you know, for her. And they asked for family intervention therapy and family therapy. And they use to come to the house. My mom would cry and talk to them and they were very caring and. They helped her in ways I couldn't. I couldn't express. I couldn't talk to her because the voices were quite aggressive towards her. That intervention team and the Medical Centre they kind of did that for me and that really calmed her down and. Yeah, she trusted them. Yeah, she couldn't do that for me right now. They stepped in really well….I feel like she really stepped up. She took the time to get educated and um to know what was going on. And um, and I think the conversations she had behind the scenes with the medical teams and the therapist without me helped her to leave me alone sometimes and helped her to deal with it, and which again made the relationship better, allowed us to communicate or personal things rather than the medical stuff.” (1007)  Uhm, I like the fact she's very caring. She's a kind hearted person, I'd say, and she's just not judgmental. And yeah, we get along really well… Uhm, well, I'd say like when I was not doing too great like a year or two ago and I think like she played like a really important role in helping me get better. Like to get help and she was like really patient with me and I think that really helped. Yeah, I think it could be and it can be quite difficult when someone is going through like a mental health problem. I think it can like put a strain on a relationship, but she was really good at, sort of like diffusing this strain. If you know what I mean. (1057)  “Uh, I say it's a fairly good one. We're pretty close. We do a lot of things together. Spend a lot of time together. Uhm, I talked to about everything that I need to, especially after I got ill. I kinda got. She's my first go to UM. And. Yeah, sometimes we disagree on things obviously, but I think that's normal and yeah, we, I say we've gotten a lot closer since I got ill. And I think she understands me a bit better as well, and she's a lot more patient with me. Uhm, I don't know if I can say the same about me being patient with that, but yeah, she's definitely more patient with me and she's… honestly, she's literally been my rock since I wasn't well. She gives me so much good advice now. And I think that probably comes from the fact that she knows more about what was going on with me. So she's definitely done heavy search and yeah, she's been there to support me through everything.” (1023) |
|  | **Fear of being rejected**  “I thought she would reject me or I would get kicked out of the house or you know things um I would be blamed for, but she understood it was a medical thing and it wasn't my fault. She was a caring mother in that sense.” (1007)  “Cos I didn't know what was happening with me and it's like I do want to speak about and I was too frightened to shamed. And, you know, didn't know what was going on in my head and I going mad, right? So I said to them, sort of like not to tell any relatives or anything except my brother. My brother knows, right? And I think the fact is that they I think they spoke to each other about it and then sort of like came with, you know and listen to that decision of mine, right? Not to tell the external family or anything, 'cos I don't like anybody knowing my business. Anyway, yeah and I don't want other people judging me. Or you know, there's enough when I'm on the tube or trains or buses. People looking at me and I just feel that they're constantly judging me.”(1007) |
|  | **Open dialogue and communication**  “Uh I like that we’re both understanding of each other and we communicate a lot better with each other, especially like we had family psychology sessions. So after that, it’s improved a lot. Like we understand each other, we don’t, you know, get angry at each other or shout at each other. We do it in a civilised way and we communicate it.” (1014)  “Uhm? I like that she listens. I like that she understands. I like that she went to the doctor to seek medical help. And even when I tell her about alternative therapies like acupuncture or herbal medicine, she's open to it. She understands, she takes me to my appointments, she gets medicine I need, so it's very calming and it's very reassuring that um she's there in like anytime I would say, you know, I'm gonna try this so this therapy or I need to talk to this doctor. She's very understanding, she's like straight away, Let's go to the doctor let's make the appointment the UM, Work around your work. She's. Yeah, she's very, very good at that.” (1007)  “ would like to talk about more personal things instead of organization or the logical stuff. I would like to have, um a more personal conversation with her and it, because of the psychosis I'm not, I haven't been able to do for the past 2 1/2 years have a conversation so that's something I would want…..Uhm… I would be more caring and more loving and supportive and being there for her rather than her always being there for me. I would um be able to reciprocate those um those conversations, and I know she wants to talk to me about that stuff and I'm having to shut it down or leave the room and. So that makes the guilt difficult. Talking about family and relatives or You know just talking about. Yeah, just talk about family and you know asking about her work and her friends and talking about things on TV. Or you know, just normal things that I want to do in my life. I can't have those conversations with her. That brings… makes it difficult”. (1007)  “r I wouldn't say the relationship's changed, but I say the conversations and things you talk about has changed because there's a bit more about mental health that we're talking about. or I think my dad was having some psychology sessions - I don't know. It was a psychologist..I don't know, he was talking to someone from the services. It might be the psychologist about some of the things that was going on and then we had some group psychology sessions as well with the family. So those kind of things fell into the conversations we were having. I don't think the relationship's changed so much. I think it's probably similar.” (1123) |
| **3. Boundaries and Independence** | **Acceptance about carer**  “Not really, no. I think we got a good relationship. Yeah, I'll probably change that if I could, yeah (laughs) but you can't really change those things. It's not something, it's not something so significant that I'd say I want to change it. If it could happen less yeah”. (1123) |
|  | **Controlling, Intrusiveness, Protectiveness**  ‘Even with this, even this conversation and doing this, she was kind of like asking questions like why is this? Why you had this in our therapy session? And I was like no. It's a questionnaire and to help them. Just explaining what this was, just for her ease. I think she's worried that I might get riled up or something might trigger the voices. So just letting her know that the last session we had, you know it was calming, it was normal, everything was fine and it allowed her to kind of be OK with it and have that trust again, like everything was fine. So just explain. Uhm, explaining things to her. Is very helpful for the relationship for us.’ (1007)  ‘uhm, I'd say she’s always been overprotective. I'd say that's been there, UM, and when I think of the relationship, I'd say nothing much as well what what's? What would she use to do. Kind of asking you loads of personal questions, you know we're friends and things. Um like if- if I'm on the phone to someone she’ll just come up and be like what's it about who my talking to? I get annoyed at sometimes because I don't want her to come up to me all the time and ask me what I'm doing wrong, or grow up or who am I talking to what's it about and stuff”. (1084) |
|  | **Negotiating change in relationships and arguments**  “I’ll try to speak to him in a normal way like this..like you know, when we had arguments, I wouldn’t get angry or start shouting..I will try to explain my point of view clearly, but he would just kind of ignore my point of view and just carry on like rambling about what he, what his point of view is. He wouldn’t understand where I’m coming from before. Yeah, and it was pretty like it was to me and my brother as well, it was just his personality was just like that before. Well I’d say he’s improved a lot now. I’d say, just the limitations like I didn’t see as it as it is, it wasn’t helpful at all because I thought it was just too controlling and uh, it just made me angry and it just made me not want to do what he was telling me anyway. So for like, it wasn’t, you know, it wasn’t a win win for both of us, it was more like a lose lose like nobody is getting their way”. (1014)  “I think sometimes I can be quite like there's not my mom, 'cause my mom doesn't really do anything wrong. Uhm, I don't think that she does, but sometimes I can struggle with my motivation and sometimes that gets her a bit like impatient with me in a bit angry and it definitely say that I need to work on that on my side where I need to be a bit more motivated, do more like around just like general things that around the house and stuff, because sometimes that all that dampen our relationship. If I don't listen to her sometimes. And that’s just something I need to work on my side”.(1023)  “she don't like me drinking, I have given up, some time I drink though, I'm going to stress a couple of wine glasses. Before I smoke, sometime I use cannabis with my friend. Uh, finally she is not hating, little by little she’s speaking, uh. Yeah, see, she's not pressure or anything. She says you enjoy your life but look after yourself. You are not alone in here. We all getting problem. You know she and kids she's speaking like that. She giving big freedom for me”. (1115)  “The aspect that I like is that she's you know she, UM, doesn't really question me too much about my life, about things, she's not too picky in terms of what I should be doing, what I shouldn't. And that that really. Uh, you know, helps in my in my recovery you know. It’s helping in my recovery, you know, so.’ (1061)  “ Yes yes she does- so she’ll sort of find things that are like very short right? We go for walks over and then when I had enough right we will turn back and we will head home and once I know that I'm heading home and I and I and I get happy then she she relaxes as well. So she knows that. She doesn't push me. And in the beginning in the beginning it used to be, but it's sort of change now. In the last year it has changed”. (1031)  “Uhm, I think just what I said before, like about sort of the arguments and stuff in regards to like being independent. I think it's frustrating for me because when I was at uni I got used to like having like being by myself and being a bit more independent. And then when I became unwell, of course that wasn't like appropriate or like I should not, I, you know, I should have been home. I should have been looked after, but I think since kind of getting better and UM like I've got like a job as well, like I work part time now and like and it's sort of like the age or like in my head. I'm like kind of comparing what my friends are doing, 'cos they're all graduated and they're all like doing all these things that I'm just kind of at home. And like my mom wants know where I am at all the time. And that's the bit that I don't like, which is kind of makes me sound a bit ungrateful and stuff like she's done so much for me. But I'd be lying if I said that I was like I didn't get frustrated at times”. (1010) |
| **4. Context and Influence** | **Cultural and Generational Differences**  “UM, like recently I was kind of dating someone for the first time. That's the first time, like I've like ever done that and like told my parents and stuff. Uhm, but it was just like the views about like in in regards to like relationship and like sex and that kind of thing that was just like, I just I didn't really like necessarily agree with that. I couldn't really say anything because it's like, you know, that's my mom and like I can't kind of disagree with especially when like areas like that. But it was just like some of the like judgmental like sort of tones and that kind of thing”. (1010)  “I’d say like, his intentions were always good like he, he always wanted me to like, you know, excel in life and because he used to talk to me about how hard it is for him to work like ‘cos since he didn’t get a university degree so he had to work long hours every day and said I don’t want that for you. That’s why I’m always pressuring you to do something so you can work, work an easier job like a skilled job which will pay more and you’d have to work less hours. You have more free time so he always have intentions like right. Like I understood that as well. But I’d say just the way of doing things when it came to it wasn’t right, I just couldn’t, you know, do it.” (1014)  “Uh, not really. I just think like this isn't this, doesn't have anything to do with my mental health. It might be like a generational thing, like she grew up in a different time, so she's much more traditional than I am, whereas I grew up in a different time in a more diverse society, so I think slightly differently to her so we clash on that a bit. But it's like I think we both understand that we have different views and that now that I've, you know, grown up, I've sort of developed my own views that are different to her. So I think I think that's normal. Like for every family, so.” (1057) |
|  | **Family Roles and Dynamics**  “ I think over the years like she 'cos she's learned about my situation, right? So she's more easy for her to understand now. And plus the other siblings, right? They talk amongst themselves and this sort of like more, UM? When you talk about things and you kind of like understand it more or less at ease with you. So they shared awful lot together and sometimes I'm included in that sometimes I'm not. Sometimes I choose not to be. It’s been helpful to them, like cos it’s only the one daughter lives with me. But the other two daughters they live in Stratford, which isn't that far from me, right? But they they're popping a lot so they give her, my elder daughter, the sort of like bump up that she needs as well. cos my eldest daughter we obviously live together as well”. (1031)  “Yes, she had to answer lots of questions for that. Uh, maybe because it has to do with the security, because as a boy I had, I needed less security from society, while my sister had to be look out for her safety. Because there was much more like security issues uh among South Asian woman has to face in a daily basis. I believe that was the issue. I had the full freedom and my sister had less. If I'd be honest, I believe that they know that they have discriminated. Interviewer: Sorry what I couldn't quite catch you. I believe my parents are aware that they have discriminated, but, uh, maybe they think that they have done the right thing. Like in that society in the Bengali society. Because I'm from a rural community. So I think that they did not had any choice rather than that that you give like you gave my sister a less freedom.”(1119) |
|  | **Mental Health**  “Yeah, um maybe couple of days ago. Asking where I was going after work and asking how am a spending my money and what am I spending my money on… And like things like that can be quite intrusive. And not understanding, and I can’t express it because of the voices. And yeah, you know, it seems quite psychotic. So she, yeah controlling my money, asking questions knowing it. But sometimes I give her the control, like I would hand her the money to take care of me because I know that the voices won't allow me to take care of myself sometimes, but the constant questioning makes you feel like. You know, you’re not treated as an adult in this situation”. (1007)  “Yes. Because I was feeling like, uh, I didn't have any friend, I didn't have anything to talk about or talk to, so I was creating imaginary friends like it's…… At the beginning, that was like really good like I was enjoying the moment. And eventually that turned out to be very bad idea because they had nothing to add to any conversation and they were just screaming back to me. The… what we call that… The misjudgement I did the wrong things I did. What could have been? What could have been different? So all the negative thoughts were now. Coming out through my imaginary friends. ----- Yeah, they were showing me another picture that what could in my life been if I just hold on to my sexual urges and then went for other things I desire currently, that’s it… Yes, they were being the moral police to be honest and they were just being a mirror to my mother to be honest like your life would be so much better if you just could hold on to yourself and don't be what you are right now.” (1119) |
|  | |
